# Effectiveness of a walking-focused physical exercise program for fall risk indicators in institutionalized older adults with and without intellectual disability: a pre-post quasi-experimental study

**DOI:** 10.3389/fragi.2026.1771908

**Published:** 2026-03-27

**Authors:** Liliana Andrea López Moreno, Carlos Moreno Pascual, María Consuelo Sancho Sánchez, Alejandro Moreno-Mateos

**Affiliations:** 1 Doctoral Program in Health, Disability, Dependence and Welfare, University of Salamanca, Salamanca, Spain; 2 Faculty of Nursing and Physiotherapy, University of Salamanca, Salamanca, Spain; 3 Faculty of Medicine, University of Salamanca, Salamanca, Spain; 4 Department of Sports, Salamanca City Council, Salamanca, Spain

**Keywords:** disabilities, fall risk, gait pattern, older adults, physical exercise

## Abstract

**Introduction:**

Exercise programs implemented among older adults have been extensively demonstrated to yield significant benefits; however, there is a limited body of research addressing the application of such interventions in older adults with severe or profound intellectual disabilities. This study aims to explore the effects of a structured physical exercise program on fall-risk indicators and balance-related outcomes in institutionalized older adults, both with and without intellectual disabilities.

**Methods:**

This pre–post quasi-experimental study included 56 institutionalized older adults, of whom 32 had an intellectual disability. Participants completed a 12-week structured physical exercise program targeting balance, lower-limb strength, gait pattern, and coordination. The primary hypothesis was that the program would be associated with improvements in validated fall-risk indicators within both groups.

**Results:**

Among participants with intellectual disabilities, significant improvements were observed in lower-limb strength (p < 0.001) and aerobic capacity, with a 12% increase in performance on the 6-Minute Walk Test (p < 0.001). In participants without intellectual disabilities, significant improvements were observed in balance and handgrip strength. Between-group differences were limited after adjustment for baseline values and age.

**Conclusion:**

The intervention was associated with improvements in validated fall-risk functional indicators in institutionalized older adults with and without intellectual disabilities. However, due to baseline heterogeneity and the quasi-experimental design, the findings should be interpreted as preliminary and hypothesis-generating rather than confirmatory of equivalent effectiveness.

## 1 Introduction

Physical exercise is widely recognized as a fundamental pillar for healthy aging. The World Health Organization includes physical activity among its key recommendations for older adults (World Health Organization, 2017). Various approaches and types of physical exercise programs have been implemented for this population.

The progressive aging of the population represents an increasing challenge for public health systems, particularly regarding fall prevention and the promotion of functional autonomy in older adults. Falls are recognized as one of the leading causes of morbidity, dependency, and mortality in this population, with even more severe consequences observed in individuals with intellectual disabilities, who exhibit greater physical, cognitive, and sensory vulnerability.

Individuals with intellectual disabilities are frequently institutionalized at earlier ages than the general population, a circumstance that often leads to a marked reduction in their participation in physical and recreational activities. Early institutionalization typically entails a substantial lifestyle change, characterized by diminished spontaneous mobility and limited exposure to outdoor environments that naturally promote daily physical activity. Reduced functional autonomy, coupled with the need for constant supervision and the scarcity of exercise programs specifically adapted to this population, further contributes to increased sedentary behavior. Moreover, in many residential facilities, daily routines are highly structured and tend to prioritize caregiving and assistance-oriented tasks over physical or leisure-based activities. As a result, individuals with intellectual disabilities experience an accelerated decline in motor and functional capacities, which in turn heightens the risk of falls, sarcopenia, and overall dependency.

Individuals with intellectual disabilities experience an accelerated aging process that has been extensively described in the scientific literature. Although this phenomenon does not necessarily imply an immediate institutionalization, the average age of residents in facilities specifically designed for older adults with intellectual disabilities tends to be lower than that observed in the majority of residential institutions that do not specialize in this population group. The frailty characteristic of this demographic represents a significant challenge for society, as early institutionalization may exert an accelerating effect on the decline of functional capacity. In many cases, the premature admission of individuals with intellectual disabilities into residential care is associated with their limited autonomy or reduced ability to manage activities of daily living, which in turn contributes to the decrease in the mean age of residents within these centers. In Spain, available data indicate that residences for older adults without disabilities account for 75.9% of the total, compared with 21.3% of centers serving individuals with disabilities, and 2.8% corresponding to mixed institutions. Furthermore, 84.9% of persons with disabilities residing in institutional settings are under 65 years of age (IMSERSO, 2022). There are studies (Lin et al., 2016; Heller and Sorensen, 2013) that associate physical exercise with a healthier lifestyle among individuals with intellectual disabilities, as well as with the viability of implementing such interventions within this population. These studies reinforce the need to develop and implement physical exercise programs specifically adapted for people with intellectual disabilities.

In this context, structured physical exercise programs have emerged as an effective strategy for fall prevention in older adults. Among these interventions, programs focusing on optimizing gait patterns and strengthening essential physical capacities—such as muscular strength, postural balance, and cardiovascular endurance—have demonstrated positive outcomes in both older adults with and without disabilities (Canning et al., 2015; Cadore et al., 2013). Nevertheless, the scientific literature remains scarce regarding the specific effectiveness of such interventions in individuals with intellectual disabilities. Physical exercise programs are beneficial for the prevention of falls and the reduction of fall-related indicators among older adults (Alcazar et al., 2018). Strengthening exercises targeting the lower limbs and stabilization of the trunk musculature enable older adults to maintain a more stable center of gravity, thereby reducing postural sway and improving overall balance control. Strength-oriented training performed with rapid execution of movements allows older adults to react more effectively to potential fall risk situations, enhancing neuromuscular responsiveness and protective reflexes (Hardy et al., 2007). Moreover, exercises designed to improve gait patterns enable program participants to experience simulated scenarios that prepare them to engage in autonomous movement within a controlled environment.

The physical exercise has been extensively studied as a means to improve fall risk indicators and reduce fall incidence (Papalia et al., 2020; Sherrington et al., 2017). The progressive loss of gait capacity is determined by a self-reinforcing process involving sarcopenia and the consequent decline in musculoskeletal strength, along with osteopenia, reduced aerobic capacity, chronic pain, and alterations in balance and coordination. These interrelated factors collectively contribute to the deterioration of functional mobility and an increased vulnerability to falls in later life. The individuals with intellectual disabilities experience a higher rate of falls compared to the general population (Smulders et al., 2013). Moreover, in this population, fall risk indicators such as balance or muscular strength do not appear to be directly associated with fall occurrence (Oppewal et al., 2014). Fall prevention programs typically do not specifically address the needs of individuals with intellectual disabilities, as highlighted in the 2015 Falls Free® National Fall Prevention Action Plan in the United States (Camero et al., 2015) and in protocols issued by public agencies in Spain (Subdirección General de Gestión, 2015; Ministerio de Sanidad G. de E., 2020).

This study aims to assess the efficacy of a structured physical exercise program in improving fall-risk indicators and improving balance-related outcomes among institutionalized older adults with and without intellectual disabilities. It is hypothesized that an intervention targeting gait pattern optimization and the development of muscular strength is expected to improve validated fall-risk indicators and functional autonomy in this population.

## 2 Materials and methods

### 2.1 Participants

**FIGURE 1:**
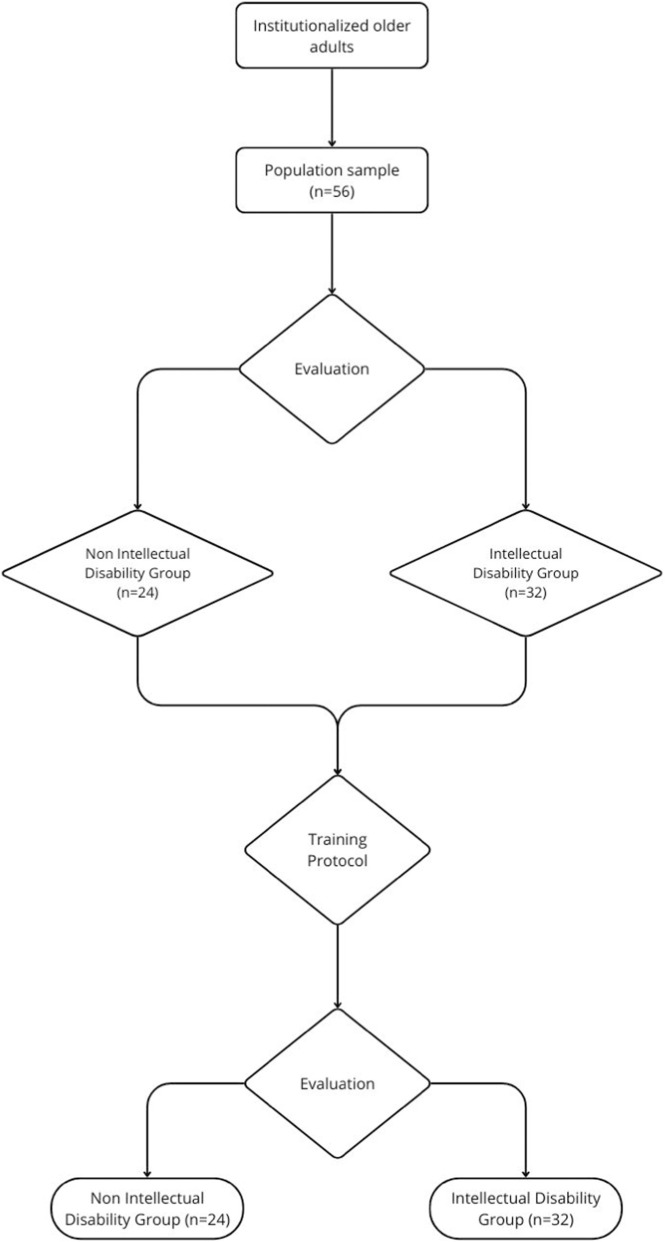
Study flow diagram. Source: Author’s own work.

Participation in the program was offered to institutionalized older adults residing in retirement homes, within the framework of a physical exercise program, regardless of sex, provided they were autonomous and independent. Older adults who expressed interest in participating and met the established inclusion criteria—being residents of the center where the program was implemented and were able to ambulate independently in an upright bipedal position, with or without orthotic assistance—and who did not meet any of the exclusion criteria (the presence of any disabling pathology that contraindicated physical exercise, absolute contraindications to exercise, and/or failure to attend the evaluations) were considered eligible for inclusion. Of the 244 residents from the institutionalized centers, 56 individuals met the inclusion criteria—32 residents with disabilities and 24 without disabilities. Attendance was mandatory, and all participants completed all sessions. Attendance was 100%, with no dropouts or withdrawals. Informed consent was obtained, and approval was granted by an ethics committee, requested from the participant or, if applicable, from their representative. The final sample included a total of 56 individuals. The consideration of the presence or absence of intellectual disability followed the American Association on Intellectual and Developmental Disabilities (AAIDD) (Schalock et al., 2021) and the legal definition established in Spain (Ministerio de Derechos Sociales y Agenda, 2030, 2023). There were 32 participants without intellectual disabilities comprised the remaining 24 participants (Figure 1). Given the marked baseline age differences between groups, between-group comparisons should be interpreted as exploratory. The primary objective of the study was to assess within-group pre-post changes following the intervention.

Assessment and Materials–Two assessments were conducted: one baseline and one post-intervention. Baseline data were collected 1 week before the start of the intervention, and outcome data were collected 1 week after the completion of the intervention. The assessments included an anamnesis, and functional evaluations were conducted by a physiotherapist in an open-plan room without elevation changes. Functional assessment was carried out in accordance with the Senior Fitness Test protocol (Rikli and Jones, 2012), including the following components: back scratch test, sit and reach test, 6-min walk test, chair stand test, Tinetti test, Timed Up and Go test (TUG), and handgrip strength using a JAMAR Plus+ dynamometer (Performance Health, Notts, United Kingdom). Data analysis was performed by a physical educator who had no direct contact with the participants, and the study was supervised by two medical doctors.

### 2.2 Intervention

The intervention lasted for 12 weeks, with three 45-min sessions per week, totaling 36 sessions. Sessions were held on Mondays, Wednesdays, and Fridays in the afternoon. It was conducted in an open-plan room without elevation changes. Sessions included the following physical exercise components: lower limb strength training using bodyweight exercises, dynamic balance, coordination, and gait retraining exercises involving obstacles, turns, changes of direction, and variations in surface type. Materials such as balls, cones, weight balls, dumbbells, and chairs with backrests were used. The intervention and evaluations were conducted by a physiotherapist, a researcher from the University of Salamanca, assigned directly to the research and not linked to the center where the intervention was carried out. The intervention was designed collaboratively by a physiotherapist and a physical educator not linked to the center. No adverse events, falls, injuries, or other intervention-related incidents were reported during the intervention period.

A predefined structured protocol detailing exercise sequencing, progression criteria, and session duration was followed to ensure consistency across sessions. Each session followed a three-phase structure (warm-up, main phase, and cool-down) consistent with standard exercise program models. This systematic approach was intended to ensure methodological coherence, physiological efficiency, and the progressive adaptation of participants throughout the intervention process.

The initial phase, corresponding to the warm-up period, consisted of a series of low-intensity and gradually progressive activities, deliberately designed to avoid abrupt, uncontrolled, or high-impact movements. The core phase, or main phase, represented the central component of the intervention and was itself subdivided into two complementary sections. The first section was focused on the training and optimization of the gait pattern, while the second concentrated on strength development, primarily oriented toward the musculature of the trunk and lower limbs. The portion dedicated to gait training aimed to promote both the analytical and global practice of walking mechanics, enabling participants to experience and adapt to a wide range of walking scenarios that closely mirrored those encountered in everyday life. Furthermore, for participants who utilized orthotic or prosthetic assistance, special emphasis was placed on the correct, functional, and safe use of these supportive devices to ensure both biomechanical alignment and postural stability. This part of the session incorporated a wide variety of movement situations, including changes of direction, transitions across surfaces of different textures and inclinations, navigation of obstacles, intentional modifications of the body’s center of gravity, lateral displacements, ascending and descending movements over raised or uneven elements, and tasks that intentionally challenged static and dynamic balance. The strength-training component, forming the second subphase of the main segment, was structured following the principles of the undulating progression training model established by the American College of Sports Medicine (ACSM) (American College of Sports Medicine, 2009). The program was designed to begin with a relatively high training volume, defined as the total number of repetitions or sets performed for each exercise, and to progressively reduce this volume as intensity levels increased. Weekly progression was individualized based on participants’ functional capacity. In addition to the lower-limb and trunk-focused exercises, specific upper-limb strength exercises were incorporated to reinforce the “parachute effect”. Finally, the cool-down or recovery phase was designed to facilitate the gradual restoration of physiological equilibrium following the exertion of the preceding phases.

The intervention sessions were conducted in person. Exercises involving movement that presented difficulty—such as abrupt changes of direction, movement over unstable elements, or modifications of the bipedal stance—were performed individually with support from the physiotherapist. The remaining exercises were performed individually by each participant simultaneously, under the supervision of the physiotherapist.

### 2.3 Statistical analyses

IBM SPSS Statistics software, version 24 (SPSS 24.0.0.0, IBM Corporation, New York, NY, United States) was used for all analyses. Descriptive statistics, including means, medians, standard deviations, variances, and minimum and maximum values, were calculated. Normality was assessed using the Kolmogorov–Smirnov test with Lilliefors correction, which indicated a non-normal distribution; therefore, non-parametric tests were applied. Group homogeneity at baseline was analyzed using the Kruskal–Wallis H test. To assess the balance between groups, Standardized Mean Differences (SMD) were calculated for continuous variables and differences in proportions for categorical variables. SMD values were interpreted as small (0–0.2), medium (0.2–0.5), and large (≥0.5). Pre- and post-intervention differences were evaluated using the Wilcoxon signed-rank test, including subgroup analyses according to the presence of musculoskeletal and neurological disorders. Effect sizes for Wilcoxon tests were calculated using Rosenthal’s r. Given the non-parametric distribution of the data, post-intervention outcomes were further analyzed using Rank ANCOVA, with baseline values, age, and group included as covariates. This approach allowed evaluation of group membership effects while adjusting for baseline differences and potential confounders. Effect sizes for Rank ANCOVA were calculated using partial eta squared (η^2^p), interpreted as trivial (0–0.10), small (0.10–0.30), moderate (0.30–0.50), and large (≥0.50). A significance level of α = 0.05 and 95% confidence intervals were adopted. No *a priori* power calculation was performed due to the pragmatic quasi-experimental design. Consequently, the study should be considered exploratory and may have been underpowered to detect small-to-moderate between-group differences, particularly given the total sample size (n = 56) and marked baseline heterogeneity. No formal correction for multiple comparisons was applied due to the exploratory nature of the analyses; therefore, findings should be interpreted cautiously.

## 3 Results

The sample consisted of 41.1% men and 58.9% women, with a mean age of 74 years (SD = 11.699). A total of 26.8% of participants used some type of orthotic aid, while 73.2% ambulated independently. Intellectual disability was present in 57.1% of the sample, whereas 42.9% did not present with intellectual disability (Table 1). The sessions were conducted without interruptions or accidents.

**TABLE 1:** Characteristics of the study sample and groups.

Participant characteristics	Sample	IDG	NIDG	p-value	SMD
N	56	32	24	​	​
Age (mean and SD)	74.00 (11.699)	64.53 (3.654)	86.63 (4.271)	<0.001*	5.56
% women	58.9%	50%	70.8%	0.117	0.44
Orthotics	28%	15.6%	41.7%	0.029*	0.60
MSD	82.1%	87.5%	75%	0.227	0.32
ND	33.9%	53.1%	8.3%	0.000*	1.12
MSD + ND	34.8%	53.6%	5.6%	<0.001*	1.23
ID	​	​	​	​	​
Severe Very severe	​	7 25	​	​	​

IDG, intellectual disability group; NIDG, Non intellectual disability group; MSD, musculoskeletal disorders; ND, neurological disorders; ID, intellectual disability; SMD, standardized mean difference

*A significance level of α = 0.05.

The study was highly heterogeneous in terms of age, the participants without intellectual disabilities (NIDG)(n = 24) having a mean age of 86.63 years and the participants with intellectual disabilities (IDG) having a mean age of 64.43 years, showing a statistically significant difference (p < 0.001). This considerable age disparity highlights the heterogeneity between the participants and should be taken into account when interpreting the results. The IDG presented a balanced gender distribution (50% men and 50% women). Regarding the use of orthotic aids, 15.6% of participants required assistance, and musculoskeletal disorders were reported in 87.5% of the sample.

In the IDG, indicators of fall risk showed moderate improvements as measured by the TUG. Substantial gains were also observed in lower limb strength, as assessed by the Chair Stand Test, and in handgrip strength. No significant changes were detected in the Back Scratch test. Regarding aerobic capacity, evaluated through the 6 Minute Walk Test, large post-intervention improvements were identified. Among participants with musculoskeletal disorders, significant improvements were found across all tests except for the Chair Stand Test and the Sit and Reach test (Table 2).

**TABLE 2:** Means and SD of disability group.

IDG	E-1	E-2	p-value	r
BackScratch test	40.59 (14.473)	40.16 (14.756)	0.131	0.26
Sit and reach test	17.19 (9.334)	17.13 (9.227)	0.817	0.04
6-min walk test	379.69 (131.284)	428.13 (123.090)	<0.001*	0.82
Chair stand test	11.75 (6.000)	13.31 (6.563)	<0.001*	0.65
Tinetti test	20.72 (4.706)	21.13 (4.791)	0.105	0.28
Up & go test	24.31 (14.702)	22.94 (14.368)	0.012*	0.44
Handgrip test right	18.5843 (6.63777)	20.4562 (6.7953)	<0.001*	0.47
Handgrip test left	19.5000 (8.5086)	20.7343 (7.5666)	0.007*	0.66

E-1, baseline; E-2, results; r, rosenthal’s r.

*A significance level of α = 0.05.

The NIDG had a mean age of 86.63 years (11.699) and included 70.8% women. In this group, 41.7% of participants used orthotic aids, and musculoskeletal disorders were reported in 75% of cases.

Within the NIDG, a significant improvement in validated fall-risk markers was observed according to the Tinetti test. Significant improvements were also identified in handgrip strength and in the Back Scratch test, although the latter showed only medium effect sizes. Aerobic capacity, as measured by the 6-Minute Walk Test, demonstrated large improvements following the intervention. Among participants without musculoskeletal disorders, no significant changes were detected, except in the Tinetti test (Table 3).

**TABLE 3:** Means and SD of NIDG.

NIDG	E-1	E-2	p-value	r
BackScratch test	37.83 (16.134)	38.88 (14.057)	0.005*	0.30
Sit and reach test	19.71 (6.969)	20.21 (6.776)	0.372	0.18
6-min walk test	410.42 (173.818)	447.92 (182.661)	<0.001*	0.71
Chair stand test	16.75 (7.344)	17.54 (7.553)	0.141	0.57
Tinetti test	22.50 (3.765)	24.13 (3.567)	<0.001*	0.75
Up & go test	13.88 (4.730)	14.92 (5.763)	0.070	0.36
Handgrip test right	16.775 (8.19964)	17.61666 (8.4025)	0.026*	0.69
Handgrip test left	16.4833 (8.30929)	17.2958 (8.4687)	0.001*	0.45

E-1, baseline; E-2, results; r, rosenthal’s r.

*A significance level of α = 0.05.

The variables showed significant associations between baseline values and post intervention outcomes, indicating that initial performance was a strong predictor of post intervention results. The Rank ANCOVA analyses (Table 4) included baseline values, age, and group as factors, allowing the effect of each variable to be evaluated independently of the others. Rank ANCOVA analyses adjusted for baseline values and age showed that, overall, no significant differences were observed between IDG and the NIDG in most post intervention functional variables. However, the Tinetti test revealed statistically significant differences between groups (p = 0.020), indicating a moderate group effect after adjusting for covariates. In contrast, all tests showed significant associations between baseline and E-2 scores (p < 0.001), confirming that initial performance was a strong predictor of post intervention outcomes, whereas age did not demonstrate a statistically significant independent effect.

**TABLE 4:** Results of rank ANCOVA for IDG and NIDG: significance and effect sizes (η^2^) adjusted for baseline and age.

Test	p-value NIDG-IDG	η^2^	p-value baseline adjusted	η^2^	p-value age adjusted	η^2^
BackScratch test	0.921	0.00	<0.001*	0.85	0.878	0.00
Sit and reach test	0.881	0.00	<0.001*	0.81	0.714	0.00
6-min walk test	0.467	0.01	<0.001*	0.91	0.607	0.00
Chair stand test	0.960	0.00	<0.001*	0.925	0.636	0.00
Tinetti test	0.020*	0.10	<0.001*	0.87	0.170	0.03
Up & go test	0.509	0.00	<0.001*	0.831	0.801	0.001
Handgrip test right	0.914	0.00	<0.001*	0.911	0.903	0.000
Handgrip test left	0.787	0.00	<0.001*	0.924	0.267	0.024

NIDG, non intellectual disability group; IDG, intellectual disability group; E-1, baseline.

*A significance level of α = 0.05.

## 4 Discussion

The modest sample size further limits statistical precision and may increase the risk of type II error, although it is comparable to that of previous studies (Nishiguchi et al., 2015; Albinet et al., 2010; Liu-Ambrose et al., 2012; Liu-Ambrose et al., 2010), there are publications with both larger (Subdirección General de Gestión, 2015; Nocera et al., 2015) and smaller samples (Nocera et al., 2015; Albinet et al., 2016), or that other variables, such as nutritional status, were not considered in the sample selection, which could affect the results. A direct comparison between NIDG and IDG cannot be established. The inclusion of a group performing the intervention in parallel to the IDG was intended to assess the feasibility of the proposed intervention within both populations.

The implemented program addresses the need to design feasible interventions that can be executed by a multidisciplinary team within the context of the institutional setting itself, since the materials employed originate either from the field of physical education or from the furniture and equipment already available within the facility. The intervention model is characterized by being easily replicable, scalable, and extendable over time, as has been proposed for the centers in which the program has already been implemented. Proposing a simple intervention in terms of cost and resource availability ensures that participants can continue to benefit from physical exercise over the long term, rather than being limited to the duration of the structured intervention period.

The volume of the intervention is also consistent with that of other studies, such as Albinet (Iuliano et al., 2015) with 42 sessions, Albinet (Liu-Ambrose et al., 2012) with 36 sessions, Iuliano (Lü et al., 2015) with 36 sessions, and Lu (Leckie et al., 2014) with 36 sessions. However, longer interventions can also be found, such as those by Leckie (Guía de ejercicio Físico para, 2012) with 144 sessions or Liu-Ambrose (Oken et al., 2006) with 56 sessions.

The IDG, being younger and functionally stronger at baseline, showed improvements, particularly in strength and general functional capacity tests. In contrast, the older NIDG may have been more physically limited, which likely impacted their outcomes. The population profile data selected for this pre–post quasi-experimental study are consistent with those reported by the IMSERSO (IMSERSO, 2022). In our study, the mean age of the NIDG, composed of individuals without intellectual disabilities, was 86.63 years. According to IMSERSO data, 74.5% of the institutionalized population in residential care centers in Spain are over the age of 80 years, and 68.2% of these residents are women, a figure comparable to the 70.8% observed in the present study. In contrast, for the IDG, the mean age was 64.53 years. National data indicate that 84.9% of institutionalized individuals with disabilities in Spain are under the age of 65, with 58.4% being women, compared to 50% in our sample. The program was associated with functional improvements, particularly in participants with higher functional vulnerability, such as those with neurological and musculoskeletal disorders. The heterogeneous responses observed highlight the importance of adapting interventions to individual needs to maximize benefits.

The IDG showed significant improvements in functional tests following the exercise program, particularly in dynamic balance tests such as the TUG and the Chair Stand test, consistent with findings from gait retraining studies (Blyth and Noguchi, 2017; Buchner et al., 1997; Sherrington et al., 2011; Dibrezzo et al., 2005). These gains, more pronounced in participants with concurrent neurological disorders, may reflect a previously sedentary baseline, as reported in other research (Ortega-Bastidas et al., 2023). This has clinical relevance as it reduces fall risk indicators and may contribute to improved functional capacity in older adults, both with and without disabilities. Although the study showed significant improvements in gait parameters, the relatively small sample size may have limited the statistical power to detect smaller differences between groups. Future studies with larger samples are needed to confirm these findings and to ensure sufficient power for the observed effect sizes.

The NIDG showed better results in the Tinetti test, which includes both static and dynamic balance assessments (Motl and McAuley, 2010), though no significant improvements were seen in dynamic balance-specific tests. Gait patterns may be significantly altered by underlying pathologies or already established, limiting technical improvements. The NIDG’s significant improvement in the Back Scratch test is consistent with findings in other studies for older adults (Bidaurrazaga-Letona et al., 2023; Mason et al., 2016; Sousa et al., 2014).

Regarding aerobic capacity as assessed by the 6-min walk test, both groups showed significant performance improvements, suggesting that the gait-focused intervention program is a beneficial means of improving aerobic capacity, as supported by other studies (Toraman et al., 2004; Taguchi et al., 2010; Michael et al., 2009). These gains could be due to the frequent displacement and continuous technical gait improvement integrated throughout the intervention, as suggested by Severinsen (Severinsen et al., 2014). Medication use may have negatively affected test performance, as previously observed (Severinsen et al., 2014), although further studies are needed to confirm this relationship (Clarke and Witham, 2017).

In the meta-analysis by Papalia et al. (2020), it was concluded that programs including postural control and strength exercises improve both dynamic and static balance. However, Granacher et al. (Peron et al., 2011) argued that strength and balance are independent factors. Some fall prevention programs focus on environmental adaptations and patient education (Granacher et al., 2012; Cumming et al., 2008; Becker et al., 2003), while the intervention in this study included balance training, as seen in other research (Cumming et al., 2008; Becker et al., 2003; Dyer et al., 2004; Donald et al., 2000; Faber et al., 2006; Rosendahl et al., 2008), as well as strength training (Becker et al., 2003; Dyer et al., 2004; Jensen et al., 2003; Donald et al., 2000; Faber et al., 2006; Sakamoto et al., 2006; Nowalk et al., 2001). Functional improvements were observed in both groups; however, due to baseline heterogeneity, comparative effectiveness cannot be definitively established. These findings suggest that structured gait-focused exercise may be feasible and associated with functional gains in institutionalized older adults with intellectual disabilities, although equivalence of response between populations cannot be established within the present design.

The significant associations between baseline and post intervention values reinforce the importance of initial functional status as a predictor of progress following training. Consequently, the intervention was associated with maintenance or improvement of functional mobility in both groups, particularly in components related to postural control and key physical capacities associated with the fall-risk markers in older adults.

Traditionally, physical exercise programs targeting older adults have primarily focused on the development of aerobic capacity and muscular strength (McMurdo et al., 2000), seeking to achieve improvement through physical conditioning, while often neglecting the technical components of gait movement. In the field of high-performance sports, it is well established that training the technical aspects of movement contributes significantly to improved performance in competition (Nelson et al., 2007). However, in many instances, exercise programs designed for older adults—with or without intellectual disabilities—omit the inclusion of activities aimed at improving or restoring gait technique. A deficient gait pattern can substantially increase the risk of falls, as it is often associated with inefficient postures and repetitive maladaptive movements that, over time, may lead to myalgias or musculoskeletal discomfort resulting from poorly executed motor gestures (Issurin, 2017).

A limitation of the study is that the lack of homogeneity between groups does not allow for concluding that the exercise program implemented in the intervention is more effective, specifically for individuals with intellectual disabilities. Nevertheless, it provides preliminary support for the feasibility and responsiveness of the intervention in the IDG. This finding suggests that an exercise-based intervention designed to improve gait patterns can generate positive outcomes in individuals both with and without intellectual disabilities, without the need to adapt the intervention solely based on the disability. The quasi-experimental pre–post design does not allow causal inference; observed improvements may reflect non-specific effects such as increased attention, test familiarity, or natural variability.

The substantial baseline heterogeneity between groups, particularly in age (SMD = 5.56) and baseline functional capacity, represents a major methodological limitation. Although statistical adjustment using Rank ANCOVA was applied, such extreme imbalance cannot be fully controlled analytically, and residual confounding is highly probable. Therefore, between-group comparisons should be interpreted with caution, and the primary interpretation of findings should focus on within-group pre–post changes. Such heterogeneity is largely attributable to the earlier institutionalization of older adults with intellectual disabilities (Jia et al., 2024; Dixon-Ibarra et al., 2013; de Winter et al., 2012), a phenomenon previously described in the literature, in contrast to the typically later institutionalization of older adults without disabilities (Hilgenkamp et al., 2012; Mulasso et al., 2015; Dyer et al., 2023). Extending the program over a longer period would be necessary to observe long-term outcomes, thereby enhancing the validity and reliability of the intervention. Sustaining the intervention would also allow its implementation across a greater number of centers and participants, facilitating broader generalization of the findings. Additionally, future analyses could incorporate self-perceived outcomes reported by participants, particularly regarding autonomy and the ability to perform activities of daily living. Furthermore, the results of the present study were inevitably influenced by external factors that may have affected the intervention’s impact, such as participation in other therapeutic programs, self-perception, individual lifestyle habits, and other contextual variables. The absence of a non-intervention control group limits causal inference. Observed improvements may partially reflect learning effects, increased test familiarity, Hawthorne effects, or regression to the mean. Future randomized controlled trials are needed to confirm efficacy. The absence of an *a priori* power calculation further limits the ability to determine whether the sample size was sufficient to detect smaller effect sizes. Finally, the study was limited by the lack of access to participants’ fall incidence data, as this variable was not controlled by the researchers. The absence of statistically significant group effects in most adjusted analyses should not be interpreted as evidence of equivalence, but rather as an indication that no clear statistical separation between populations could be demonstrated under the present design constraints.

In conclusion, the intervention aimed at improving gait patterns was associated with enhancements in walking capacity, as observed in the 6-Minute walk test, and potential improvements in validated fall-risk markers and risk indicators. Functional improvements were observed in participants both with and without intellectual disability; however, due to the quasi-experimental design and baseline heterogeneity, the findings should be interpreted as preliminary and hypothesis-generating rather than confirmatory of equivalent effectiveness. However, given the pre–post quasi-experimental design, the absence of randomization, and baseline heterogeneity between groups, further studies with longer interventions, more homogeneous samples, and larger participant numbers are required to establish causal relationships. Future lines of research should incorporate variables such as medication use and fall incidence, as well as include a larger study sample that allows for greater generalizability of the results to the broader population, achieved through the involvement of a greater number of centers.

The authors declare that the research was conducted in the absence of any commercial or financial relationships that could be construed as a potential conflict of interest.

## Data Availability

The datasets presented in this article are not readily available because the dataset is not accessible due to personal data protection reasons, in accordance with local legislation. Requests to access the datasets should be directed to amoremom@usal.es.
